# Spatially and temporally continuous LAI datasets based on the mixed pixel decomposition method

**DOI:** 10.1186/s40064-016-2166-9

**Published:** 2016-04-26

**Authors:** Jianjun Zhao, Yanying Wang, Hongyan Zhang, Zhengxiang Zhang, Xiaoyi Guo, Shan Yu, Wala Du

**Affiliations:** School of Geographical Sciences, Northeast Normal University, Changchun, 130024 Jilin China; Inner Mongolia Key Laboratory of RS and GIS, Huhhot, 010022 Inner Mongolia China; Ecological and Agricultural Meteorology Center of Inner Mongolia Autonomous Region, Huhhot, 010051 Inner Mongolia China

**Keywords:** AVHRR, MODIS, LAI (leaf area index), NDVI, Mixed pixel decomposition, Empirical model

## Abstract

The leaf area index (LAI) is a key biophysical parameter that determines the state of plant growth. A global LAI has been routinely produced by the Moderate Resolution Imaging Spectro-radiometer (MODIS) and Advanced Very High Resolution Radiometer (AVHRR). However, the MODIS and AVHRR LAI products cannot be synchronized with the same spatial and temporal resolution. The LAI features are not discernible when a global LAI product is implemented at the regional scale because it has low resolution and different land cover types. To obtain high spatial and temporal resolution of LAI products, an empirical model based on the pixel scale was developed. The approach to generate a long (multi-decade) time series of a 1-km spatial resolution LAI normally integrates both AVHRR and MODIS datasets for different land cover types. In this paper, a regression-based model for generating a vegetation LAI was developed using the AVHRR Global Inventory Modelling and Mapping Studies Normalized Difference Vegetation Index (NDVI), MODIS LAI and land cover as input data; the model was evaluated by using relevant data from the same period data from 2000 to 2006. The results of this method show a good consistency in LAI values retrieved from the AVHRR NDVI and MODIS LAI. This simple method has no specific-limited data requirements and can provide improved spatial and temporal resolution in a region without ground data.

## Background

The leaf area index (LAI) is a key parameter of models and has been widely applied to study vegetation, hydrology, ecology and climate change. The LAI is one sided and, as such, is one half the green leaf area when both sides of leaves are considered. The LAI is the leaf surface area per unit ground area (Shabanov et al. 2005).

Conventional ground-based measurement data are restricted by spatial and temporal scales, and the field measurement of the LAI is difficult to obtain for a large area. Remote sensing technologies could cover the gap. Therefore, it is critical to obtain a high-quality long time series LAI from remote sensing data to obtain practical solutions.

There are two types of methods for estimating the LAI using satellite sensors (Deng et al. 2006). The first type is based on vegetation indices (VIs), i.e., various combinations of reflectance in different spectral bands. In addition to the most often used VIs, such as the Normalized Difference Vegetation Index (NDVI) (Rouse et al. 1974) and simple ratio (SR) (Jordan 1969), a large number of other indices (Huete 1988) have been used to relate LAI to surface reflectance (Liu et al. 2007).

Many scholars use Advanced Very High Resolution Radiometer (AVHRR) data for global and regional studies, and some good theoretical methods have been provided for LAI production. Chen et al. (2002) used high-resolution satellite data and ground measurements data to calculate the coarse-resolution LAI. Deng et al. (2006) applied an algorithm to generate the global retrieval of the LAI (Deng et al. 2006). Liu et al. (2007) adopted a 4-scale model to estimate the AVHRR LAI. Tang et al. (2007) developed a new algorithm using the principles of ground measurements LAI based on the canopy gap fraction to calculate LAI. Ganguly et al. (2008a, b) built a physically based approach to produce a long (multi-decade) time series of LAI data. These LAI results have deficiencies when used at the regional scale (Deng et al. 2006; Liu et al. 2007). In the global LAI research, some regions of continents were taken as representative to calculate the LAI. These methods cannot reflect the LAI features for different time durations and vegetation types in distinct places. With 8-km special resolution, the LAI produced is hard to use to satisfy the need for regional research. Therefore, it is necessary to study suitable algorithms that can produce a long time series LAI by combining high temporal and spatial resolution data.

In many studies (Chen et al. 2002), the LAI in the algorithm was estimated by considering the relationships between LAI and VIs from remote sensing data (Deng et al. 2006). In this paper, a new LAI dataset was calculated by using NDVI–LAI models that combine Global Inventory Modelling and Mapping Studies (GIMMS) and Moderate Resolution Imaging Spectro-radiometer (MODIS) data (Yang et al. 2006a, b).

In this paper, the focus was on zonal coarse-resolution LAI maps in Northeast China based on AVHRR and MODIS data. As one of the main products of the MODIS sensor, the MODIS LAI (MOD15A2) has been routinely produced and is increasingly used for global and regional studies (Deng et al. 2006; Liu et al. 2007). This study developed an empirical, generalized, regression-based, regional scale LAI model by combing AVHRR data and derived products from MODIS. Then, pixel-to-pixel statistics with the AVHRR and MODIS data were utilized to set up an empirical relationship between the NDVI and LAI for different land cover types, and the AVHRR LAI was calculated for Northeast China from 1981 to 2006. The objectives of this article are as follows: (1) to explain the principle of this new algorithm, (2) to validate the algorithm, and (3) to compare the LAI result with a MODIS LAI image. Once developed, this regression model could be directly applied to other regional scales.

## Methods

### Materials

**Fig. 1:**
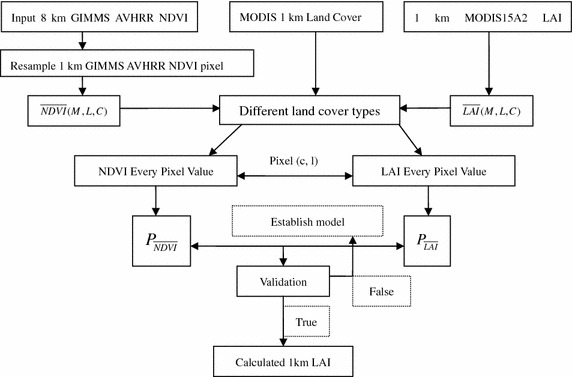
The flow chart of the algorithm

In this research, two datasets were used: GIMMS NDVI and MODIS LAI. Figure 1 describes the mechanism of the LAI retrieval approach. The MODIS LAI product was used for the overlap period from 2000 to 2006 between the two datasets in this study.

The 15-day maximum value AVHRR NDVI products (Ganguly et al. 2008a, b; Holben 1986) from the GIMMS groups from July 1981 to December 2006 were used as the input dataset (Ganguly et al. 2008b; TUCKER 2005; Chen and Wang 2009). The data have been corrected to reduce the effects from volcanic aerosols as well as other effects (Ganguly et al. 2008a, b; Tucker 2005; Brown et al. 2006; Holben 1986). The 15-day maximum-value NDVI composites were calculated as the mean value and the monthly 8-km AVHRR dataset was resampled to a 1-km spatial resolution; then, the dataset was used to produce the monthly LAI product with a 1-km resolution.

The MODIS LAI dataset was used as input data to calculate the AVHRR LAI dataset (Shabanov et al. 2005; Yang et al. 2006a, b). The monthly LAI product was generated using the average of the 8-day maximum-value composites methods (Yang et al. 2006a, b).

The land cover maps were an ancillary data input to the LAI retrieval algorithm. In the implementation of the algorithm, any land cover map can be used, but in our case, the MODIS-derived LAI/fPAR scheme land cover map (https://lpdaac.usgs.gov/lpdaac/products/modis_products_table/land_cover/yearly_l3_global_1km2/mod12q1) was adopted. Snow/ice, bodies of water, and non-vegetated and urban classes were not considered in the LAI retrieval. The algorithm refers to six land cover types (biomes) with a 1-km resolution: (1) grasses and cereal crops (biome1), (2) shrubs (biome2), (3) broadleaf crops (biome3), (4) the savannah (biome4), (5) broadleaf forests (biome5), and (6) needleleaf forests (biome6) (Yang et al. 2006a, b).

These six biomes were estimated to be 99 % of the total area. Other biomes had a high signal to noise ratio of the MODIS LAI, and some had values of more than 25. Because it was difficult to obtain the NDVI–LAI relationship, the algorithm of Chen et al. (2002) was applied in the study.

### Methodology

There are various methods for obtaining the LAI. The retrieval algorithm of vegetation structure parameters can be put into four categories of remote sensing: (1) using relationships between the LAI and VIs; (2) using the traditional optimization algorithm for the vegetation canopy model; (3) using a look-up table; (4) using a neural network.

Based on the experience of the LAI-VI model, this algorithm belongs to a statistical method, which calculates the LAI through a statistical relationship between spectral indexes.

The change rule of the LAI has a large difference in different time series vegetation types based on the LAI data products provided by MODIS. A pure pixel of the LAI can be determined with the change rule through the statistical method. However, it is hard to satisfy the requirements of the time scale because MODIS products were only implemented in 2000. AVHRR data covered 1981–2006, but the coarse resolution, with its relatively mixed pixels, needs to be decomposed to obtain higher precision pixel of the LAI values.

The traditional empirical model only uses a single VI and LAI formula to obtain the LAI. The AVHRR LAI was calculated by using the empirical model between the AVHRR NDVI and MODIS LAI in this paper, while the regional vegetation classification data were applied to reduce regional limitations of the model. Different LAI and VI empirical models were adopted for different time durations and different vegetation types. At the same time, the 8-km resolution image was decomposed into a 1-km NDVI. Its output participated in the calculation to finally obtain the AHVRR LAI.

The original AVHRR 8-km dataset was resampled into a 1-km grid. The monthly average in different years is calculated as

1 $$\overline{NDVI} (M,L,P) = \frac{1}{{N_{y} }}\sum\limits_{Y = 2000}^{2006} {NDVI(Y,M,L,P)}$$

The mean LAI is calculated as

2 $$\overline{LAI} (M,L,P) = \frac{1}{{N_{y} }}\sum\limits_{Y = 2000}^{2006} {LAI(Y,M,L,P)}$$

where *Ny* is the number of years (*Ny* = 7), *M* is month, *L* is the land cover type, and *P* is pixel.

Random errors came from pixels with mixed land cover types in this study. The radiative signals are quite different for different vegetation types at the same LAI. Accurate identification of the mixed pixels of different land types is the key to providing LAI estimation accuracy (Chen et al. 2002). Here, a method of mixed pixel decomposition was adopted, and the variation curves of different land cover types at the AVHRR sub-pixel scale were determined. The model is defined as

3 $$AVHRR_{LAI} = f_{N\_L} \left( {HM,L,AVHRR_{NDVI} } \right)$$

*AVHRR*_*LAI*_ is the LAI value of the mixed pixel decomposition, *HM* is time, *AVHRR*_*NDVI*_ is the AVHRR NDVI value, *L* is the land cover types, *f*_*N*_*L*_ is the relationship between the AVHRR NDVI, and the MODIS LAI denotes different land cover types.

During the decomposition of AVHRR data from an 8- to 1-km resolution, its value equaled the mean of the 64-sub-pixels value (or each sub-pixel value). If a 1-km resolution AVHRR NDVI and MODIS LAI were directly used to retrieve pixels one by one, the result of the retrieval of the LAI would have an 8-km spatial resolution. The result had no effect on the precision of pure pixels, but it did effect the precision of mixed pixels. There was no strong relationship between the NDVI and LAI. To improve the retrieval accuracy of resolution, all the pixels with the MODIS LAI as a benchmark were analysed, some noise pixels were removed, and the mean value at the same LAI and the mean value of the AVHRR NDVI corresponding with MODIS pixels were calculated. Then, spatial resolution was strengthened by the retrieval of the LAI. This method of analysing the mean value of the NDVI and LAI and establishing the regression equation assisted in the role of mixed pixel decomposition and greatly decreased the LAI retrieval error.

The mean value of NDVI was calculated as

4 $$P_{{\overline{NDVI} }} = \frac{1}{n}\sum\limits_{i = 1}^{n} {\overline{NDVI} (M,l,p_{i} )} \quad (1,2, \ldots ,12;\;\;l = 1,2, \ldots ,6;\;\;\;0 \le i \le n)$$

where $P_{{\overline{NDVI} }}$ is the mean value of NDVI, *M* is month, *L* is the land cover type, *N* is the number of pixels, and *P*_*i*_ is the pixel corresponding to the same location from the LAI; there is no regularity in the image.

Different regression equations, including exponential, power and linear curve equations, were found for different time durations and land cover types. The rule was the same as in previous studies, i.e., exponential > Power > Linear, but the correlation coefficients were different, and all types had linear correlation characteristics. When the algorithm was not linear, (Eq. 5) was true. Because of the nonlinearity of the algorithm, different results were obtained depending on whether the individual pixel was first averaged before the algorithm was applied. A precise description of the algorithm was given by Chen (1999).

5 $$f_{AVHRR} \left( {\overline{NDVI} } \right) \ne \frac{1}{n}\sum\limits_{i = 1}^{n} {f_{AVHRR} \left( {NDVI} \right)}$$

Because $P_{{\overline{NDVI} }}$ was calculated using the average of $\overline{NDVI}$, the linear equations were defined as

6 $$LAI(HM,L,C) = f_{{AVHRR_{NDVI} \_MODIS_{LAI} }} (NDVI(HM,L,C))$$

The retrieval index was used to control the pixels during computation. RI is the ratio of retrieved LAI pixels to the total number of pixels, which is defined as

7 $$RI = \frac{Number\;of\;retrieved\;pixels}{Total\;number\;of\;processed\;pixels}$$

The RI was used to assess uncertainties of the model. In general, the RI value increased with increasing uncertainties (Ganguly et al. 2008a, b).

RMSE is the root mean square error between the input MODIS LAI and the output AVHRR LAI. It was used to evaluate the calculation precision of the retrieved AVHRR LAI data, which is defined as

8 $$RMSE\left( {Y,M,L} \right) = \sqrt {\frac{1}{N}\sum\limits_{k = 1}^{N} {\left[ {LAI_{AVHRR} \left( {Y,M,L} \right) - LAI_{\text{MODIS}} \left( {Y,M,L} \right)} \right]^{2} } }$$

Here, LAI_AVHRR_ values were new LAI values generated by the AVHRR model of the algorithm, and LAI_MODIS_ represents the MODIS LAI values. The RMSE is a function of year, month, land cover type and image size. RMSE can accurately measure the value and the simulation model of fitting.

## Results

### Relationship between the AVHRR NDVI and MODIS LAI

In this paper, LAI algorithms were developed using AVHRR NDVI and LAI images of Northeast China from 1981 to 2006. These algorithms were based on pixels at the regional scale and vegetation indices in an empirical relationship.

**Fig. 2:**
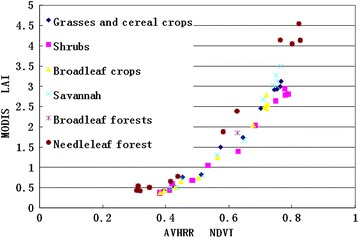
The relationship between the NDVI and LAI for different land cover types

Because the calculation of the relationship between the NDVI and LAI uses the average algorithm, the relationships between the NDVI and LAI were linear for different land cover types (Fig. 2). The relationship between the NDVI and LAI of the six land cover types is shown in Fig. 2. A saturation point at a high LAI was not included in the graph. It shows that NDVI values could be adapted for the calculation of the LAI.

**Fig. 3:**
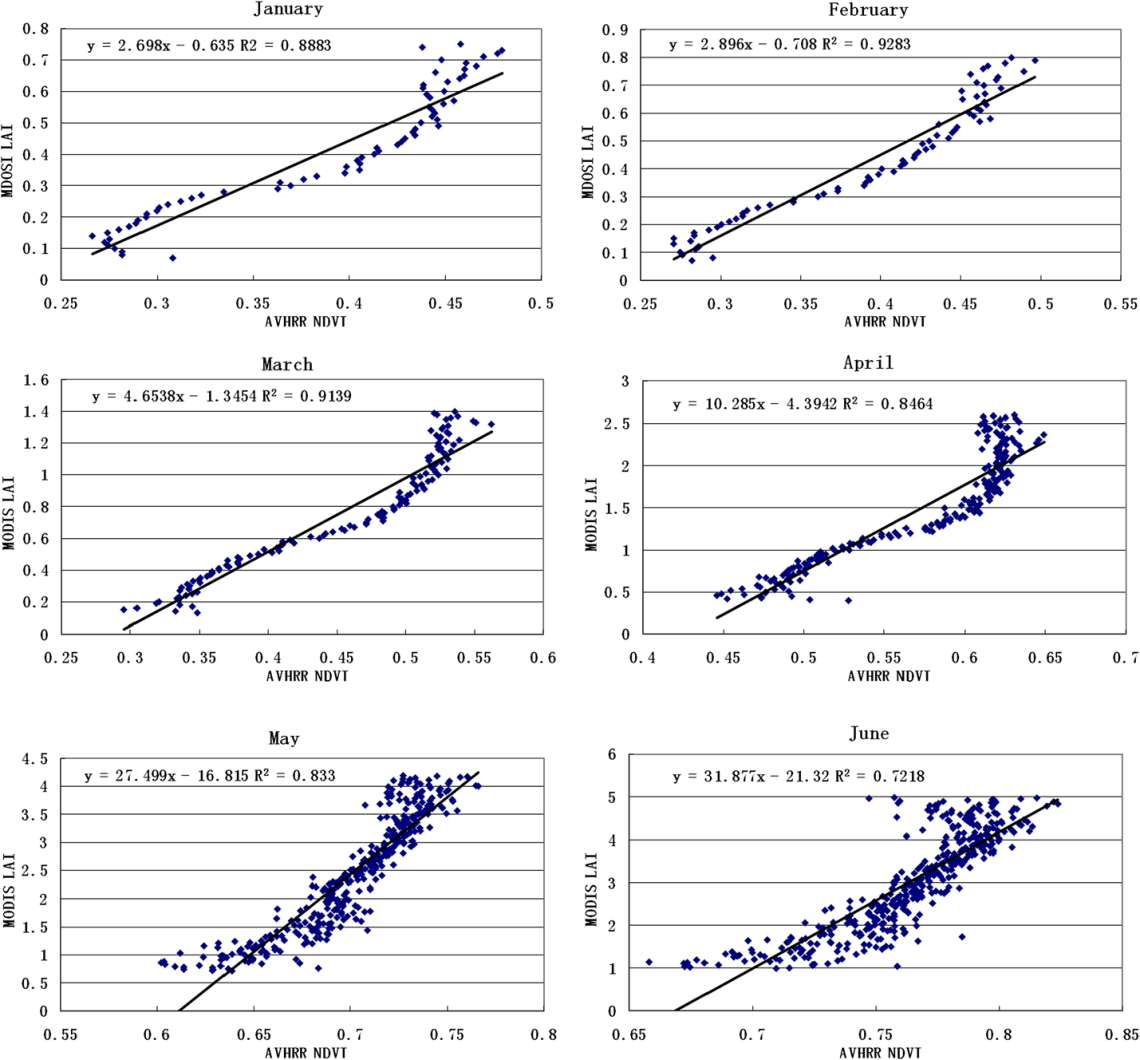
The correlation coefficient over 6 months

**Fig. 4:**
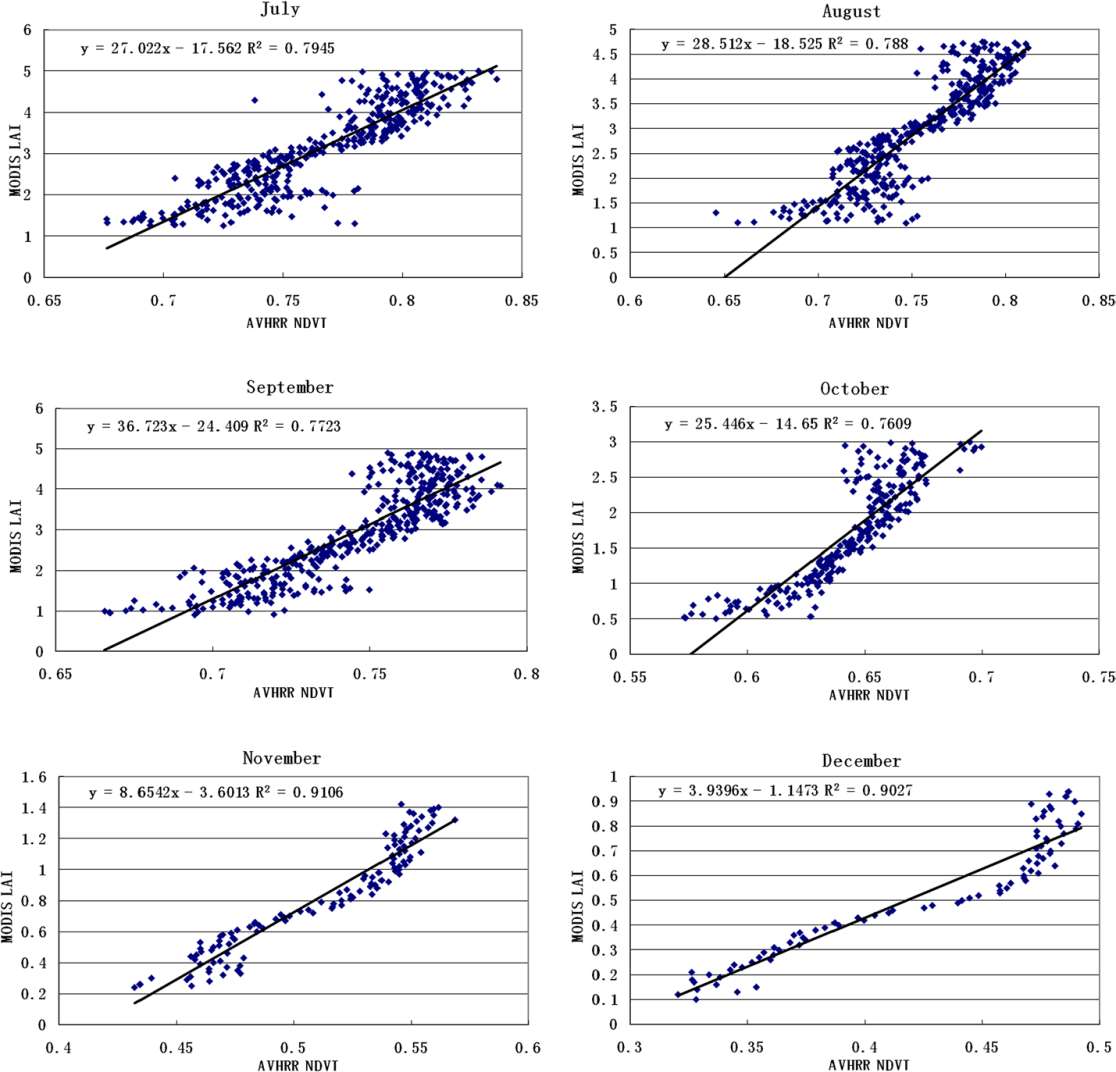
The correlation coefficient over 6 months

To analyse the relationship between the NDVI and LAI for different land cover types at the pixel scale, all land cover types over 12 months were analysed. Figures 3 and 4 presents the correlation coefficient of grasses and cereal crops, with a good correlation over 12 months.

Table 1 lists the correlation coefficients of different seasons for the land cover types. It shows a better correlation between the NDVI and LAI. Correlation coefficients changed smoothly, and the value was more than 0.5. The biggest correlation coefficient was greater than 0.9 in December, January and February, while the lowest value was approximately 0.6 in June, July and August. This result was because the value reached saturation when the vegetation canopy LAI was more than 3–4. The value weakens the correlation between the NDVI and LAI to a certain extent.

**Table 1:** The correlation coefficient in different seasons

	Biome 1	Biome 2	Biome 3	Biome 4	Biome 5	Biome 6
January	0.8883	0.7934	0.9421	0.9595	0.9055	0.7786
April	0.8464	0.7665	0.8883	0.8863	0.9115	0.808
July	0.7945	0.5068	0.7859	0.7019	0.6199	0.6819
October	0.7609	0.5261	0.8197	0.8418	0.884	0.6206

### New AVHRR LAI algorithms

The land cover map produced by a MODIS-derived LAI/fPAR scheme was used for AVHRR LAI calculations. The different land cover type NDVI–LAI relationships, which were derived from the inversion of the linear function, were then converted from the NDVI to LAI. According to the results shown in Figs. 2, 3 and 4 as well as Tables 1 and 2, the following algorithms were developed for the AVHRR.

9 $$AVHRR_{LAI} = (A \times AVHRR_{NDVI} - 1)/B$$

where *X* is the half month from January to December (*X* = 0.5, 1, 1.5, …, 12). The A and B values can be calculated for different land cover types and any time in Table 3. The Chen methods were adopted to calculate other land cover types (Chen et al. 2002).

10 $$LAI = - 1.6 \times LN[(14.5 - SR)/13.5]$$

**Table 2:** The algorithm for different vegetation types and each month’s average of the MODIS LAI value and RI

	Jan	Feb	Mar	Apr	May	Jun	Jul	Aug	Sep	Oct	Nov	Dec
Grasses and cereal crops	0.97	0.97	0.96	0.96	0.95	0.97	0.96	0.96	0.96	0.96	0.97	0.97
	0.41	0.44	0.77	1.50	2.45	3.00	3.12	2.93	2.91	1.74	0.83	0.55
Shrubs	0.90	0.92	0.90	0.89	0.90	0.91	0.92	0.85	0.88	0.89	0.89	0.89
	0.36	0.39	0.60	1.05	2.04	2.94	2.81	2.79	2.64	1.40	0.89	0.44
Broadleaf crops	0.96	0.98	0.96	0.97	0.96	0.95	0.95	0.95	0.98	0.97	0.99	0.97
	0.39	0.44	0.66	1.25	2.05	2.46	2.56	2.53	2.49	1.44	0.75	0.55
Savannah	0.95	0.95	0.95	0.95	0.95	0.93	0.94	0.92	0.94	0.89	0.94	0.94
	0.47	0.49	0.76	1.32	2.67	3.49	3.27	3.26	3.02	1.67	0.80	0.52
Broadleaf forests	0.77	0.88	0.86	0.83	0.87	0.88	0.85	0.76	0.77	0.84	0.73	0.82
	0.50	0.49	0.42	0.78	1.85	4.05	4.14	4.54	4.14	1.36	0.66	0.57
Needleleaf forest	0.83	0.94	0.93	0.89	0.76	0.86	0.86	0.89	0.84	0.88	0.93	0.91
	0.43	0.44	0.54	0.78	2.39	4.05	4.14	4.54	4.14	1.88	0.66	0.51

**Table 3:** The parameters of the equation

	Equation	R^2^
Biome 1	A = 0.0008x^5^ − 0.0264x^4^ + 0.3281x^3^ − 1.7616x^2^ + 3.2793x + 2.4192	0.99
	B = 0.0005x^5^ − 0.0158x^4^ + 0.1822x^3^ − 0.8774x^2^ + 1.3018x + 1.0122	0.99
Biome 2	A = −1E−05x^5^ − 0.0033x^4^ + 0.1023x^3^ − 0.9233x^2^ + 2.3906x + 2.2805	0.99
	B = 2E−05x^5^ − 0.0011x^4^ + 0.0175x^3^ − 0.1179x^2^ + 0.2411x + 0.2073	0.998
Biome 3	A = 0.0005x^5^ − 0.0167x^4^ + 0.2168x^3^ − 1.2163x^2^ + 2.3199x + 2.4426	0.99
	B = 0.0003x^5^ − 0.0104x^4^ + 0.1268x^3^ − 0.6532x^2^ + 1.1046x + 0.5701	0.98
Biome 4	A = 0.0005x^5^ − 0.0193x^4^ + 0.2579x^3^ − 1.511x^2^ + 3.2267x + 1.5855	0.98
	B = 0.0004x^5^ − 0.0117x^4^ + 0.1429x^3^ − 0.7551x^2^ + 1.4121x + 0.2448	0.99
Biome 5	A = 0.0002x^5^ − 0.0076x^4^ + 0.1384x^3^ − 1.0258x^2^ + 2.5696x + 1.7464	0.98
	B = 8E−05x^5^ − 0.0028x^4^ + 0.0362x^3^ − 0.2111x^2^ + 0.4735x − 0.0607	0.997
Biome 6	A = 0.0003x^5^ − 0.0101x^4^ + 0.1559x^3^ − 1.0453x^2^ + 2.5012x + 1.7268	0.98
	B = 3E−05x^5^ − 0.0011x^4^ + 0.0168x^3^ − 0.1091x^2^ + 0.2345x + 0.1777	0.96

## Discussion

### Model verification

**Fig. 5:**
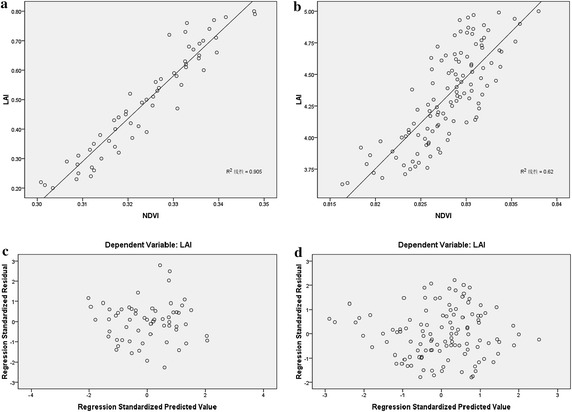
The model accuracy evaluation [Pearson correlation coefficient between NDVI and LAI (**a** and **b**), scatterplot of regression standardized predicted value (**c** and **d**)]

**Fig. 6:**
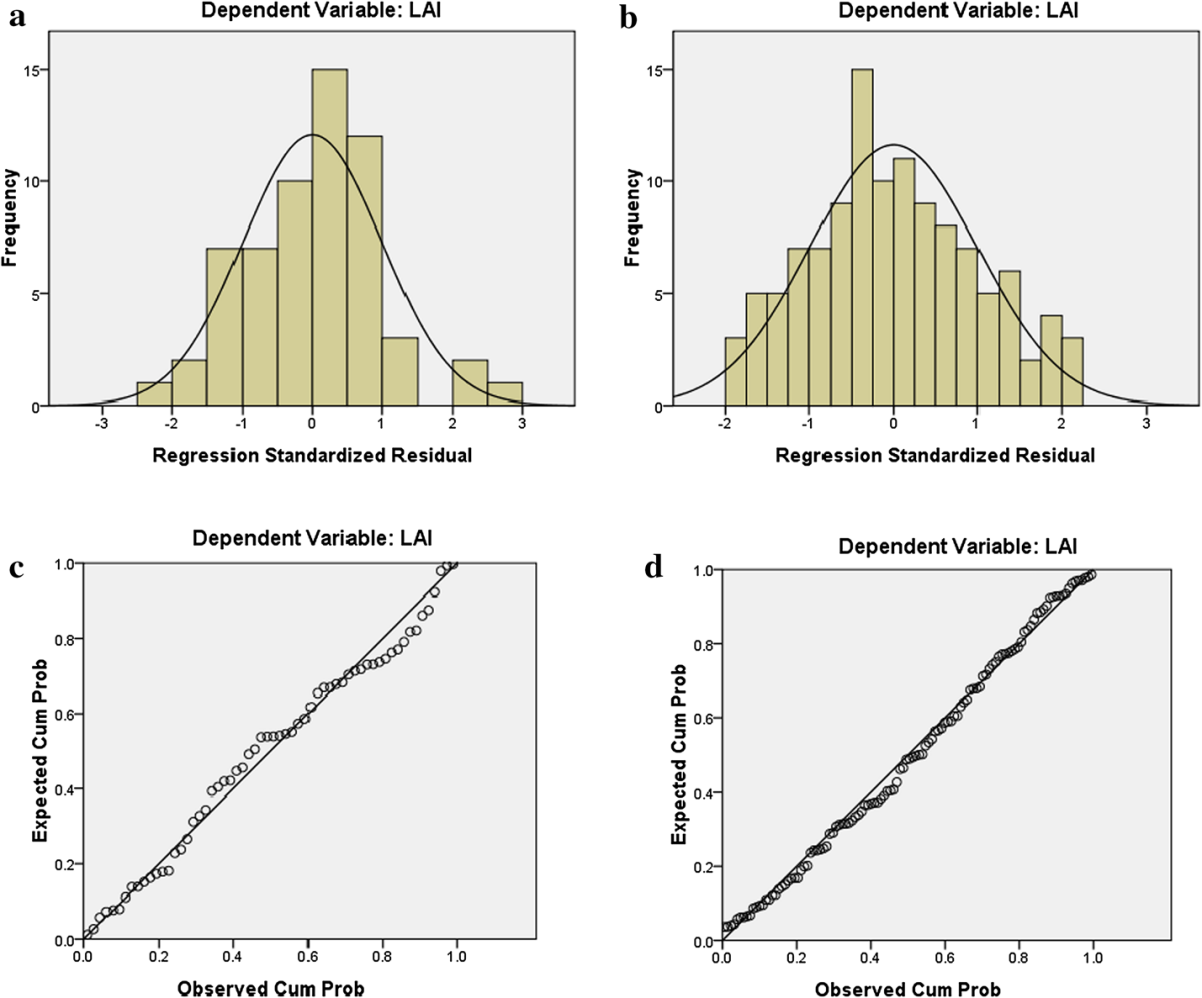
The model accuracy evaluation (histogram of regression standardized residual (**a** and **b**), normal P–P plot of regression standardized residual (**c** and **d**))

To analyse whether the linear equation was in accordance with the homogeneity of variance and normal distribution, the broadleaf forests (which take up 20 % of the total area) in two extreme months (highest in January and lowest in July) were used for testing. The results are presented in Figs. 5 and 6.

Figure 5a, b, with an R^2^ of 0.91 and 0.62, show the correlation between the NDVI and LAI for January and July. In Fig. 5c, d, the ranges of the regression standardized predicted value are (−2, 2) for January and (−3, 3) for Jul. The values are satisfactory for the homogeneity of variance. Figure 6a–d show obvious normal distributions.

### Comparison with MODIS LAI products

The algorithm was used to calculate the AHVRR LAI and then to verify the results through a comparison with MODIS LAI products of the same period.

**Fig. 7:**
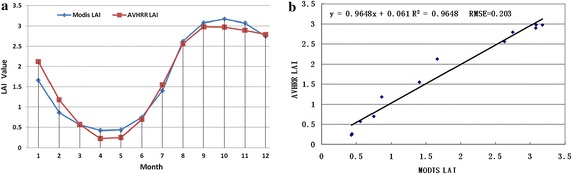
The AVHRR LAI and MODIS LAI for grasses and cereal crops types over 12 months in 2003

**Fig. 8:**
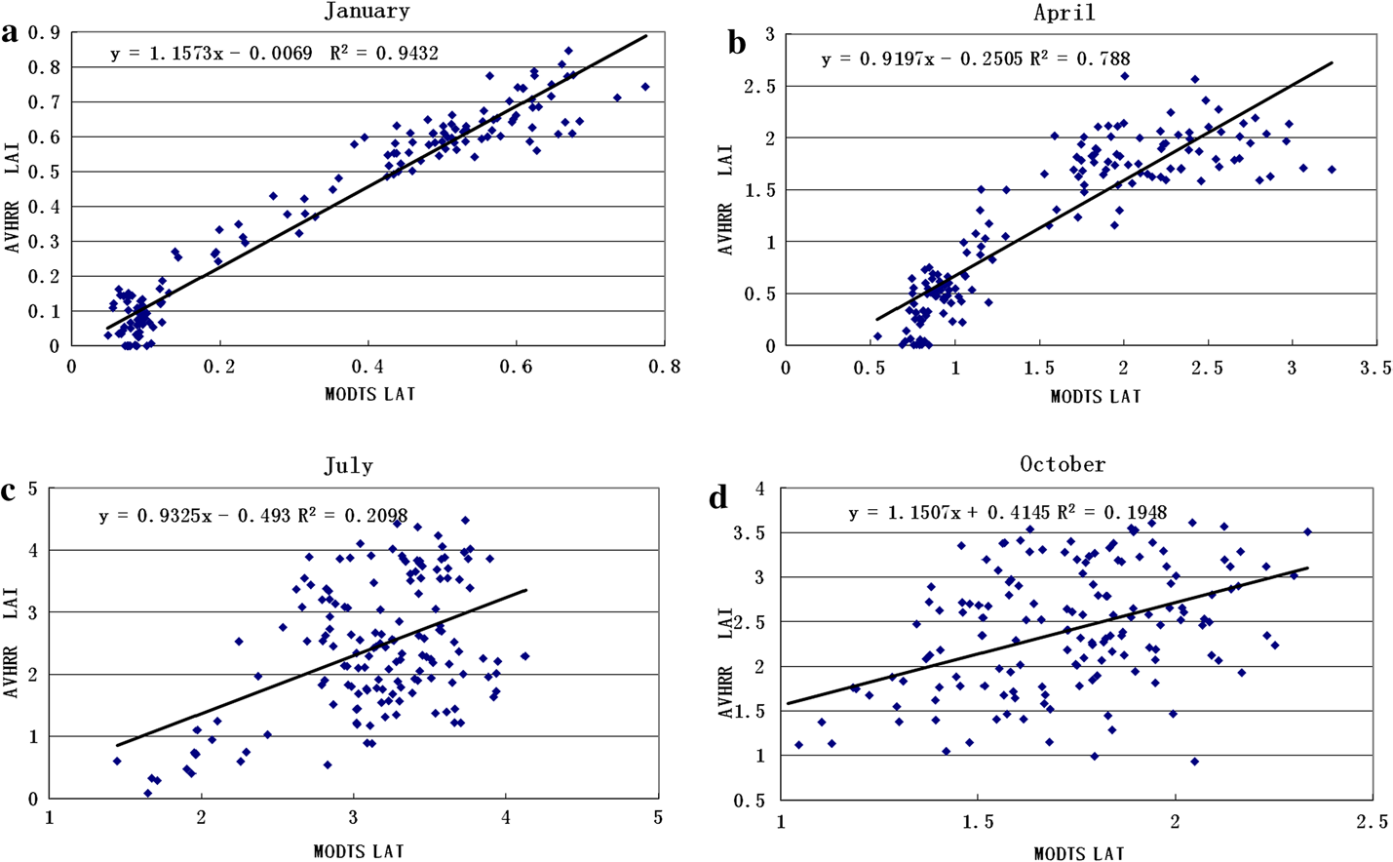
The relationship between the AVHRR LAI and MODIS LAI

A good similarity between the AVHRR LAI and MODIS LAI is displayed in Fig. 7a. The size of the LAI and amplitude values had a close relationship, while the size of fluctuations in the LAI had excellent characteristics. Their correlation coefficient was 0.96 and RSME was 0.203 in Fig. 7b. We compared the difference between the AVHRR LAI and MODIS LAI to determine the accuracy of individual pixel LAI values. The LAI values were calculated from the mean AVHRR NDVI values of individual pixels with a 1-km resolution. Matching dates (January, April, July and October) of the AVHRR LAI and MODIS LAI were selected for comparison of accuracy (Fig. 8). The scatter of points for corresponding variations of the LAI from the AVHRR and MODIS dataset was very good. There were several reasons for the scatter, including the following: (1) the errors from the two images, (2) the original resolution of AVHRR was 8 km, (3) the effect of mixed pixels and surface heterogeneity, and (4) the two images having differences in land cover types.

An assessment of the AVHRR LAI dataset in the overlapping year 2003 was performed, for which the MODIS LAI was used to obtain the AVHRR LAI algorithm. The quality of the new LAI dataset depended on the quality of the input AVHRR NDVI dataset, MODIS LAI dataset and land cover data. For each biome, all pixels over the entire year were selected for analysis. For each pixel in the images, every biome type was calculated as (Ganguly et al. 2008b)

11 $$\delta_{LAI} (i_{b} ,j_{b} ,t) = AVHRR_{LAI} (i_{b} ,j_{b} ,t) - MODIS_{LAI} (i_{b} ,j_{b} ,t)$$

where i_b_, j_b_ are pixels for different biomes, b, and t is month. Table 4 shows the accuracy ($\overline{\delta }$ is the mean value of $\delta_{\text{LAI}} (i_{b} ,j_{b} ,t)$) and precision (*σ* is the standard deviation of $\delta_{\text{LAI}} (i_{b} ,j_{b} ,t)$) of AVHRR LAI with respect to MODIS LAI for different seasons and different biomes.

**Table 4:** The LAI assessment for different seasons

Month	Biome 1		Biome 2		Biome 3		Biome 4		Biome 5		Biome 6	
	$\bar{\delta }$	σ	$\bar{\delta }$	σ	$\bar{\delta }$	σ	$\bar{\delta }$	σ	$\bar{\delta }$	σ	$\bar{\delta }$	σ
January	0.07	0.20	0.06	0.26	0.12	0.20	0.07	0.30	0.06	0.74	−0.04	0.94
April	−0.38	0.78	−0.28	0.71	−0.13	0.62	−0.41	0.91	−0.38	0.84	−0.45	1.36
July	−0.68	1.71	0.06	1.34	−0.29	1.56	−0.60	1.68	−0.28	1.64	−0.60	2.81
October	0.70	1.25	0.01	0.83	0.64	1.09	0.23	1.04	0.25	0.96	0.57	1.45

For the values of $\left| {\overline{\delta } } \right|$, the herbaceous biomes (broadleaf crops, grasses/cereal crops, savannas shrubs) were distributed in the LAI range of nearly 0–0.69 for all months, while the woody biomes (needleleaf and broadleaf forests) showed a range from 0.039 to 0.596. The AVHRR LAI was underestimated more than the MODIS LAI, especially for April and July. These larger differences indicated that new LAI retrievals from a remote sensing dataset that captured the ability of seasonality was insignificant compared to LAI retrievals from surface reflectance. The various land cover types had different precision for different seasons; January was best and July was worst, as shown in Table 4. The main reason was that the NDVI value was higher in summer and the NDVI achieved saturation; therefore, the relationship between the NDVI and LAI became weaker. Overall, the different values from the AVHRR and MODIS datasets showed that the spatial–temporal agreement and the accuracy and precision were acceptable, suggesting that the proposed computing process was successful.

## Conclusions

This research introduced an algorithm based on pixel scale for generating the LAI and its application to producing long time series of regional LAI data using MODIS and AVHRR datasets. In general, this algorithm integrated AVHRR and MODIS datasets with different temporal and spatial resolutions, and all pixels (except anomaly pixels) were utilized to calculate the related coefficients. Different algorithms were developed for deriving the LAI of different time durations and different land cover types.

The LAI algorithm presented here has desirable characteristics for regional application. (1) The models in our algorithm development were based on mathematical statistics and empirical relationships. (2) It is an effective way to regain the LAI by using the NDVI, and placing emphasis on regional scale applications that are based on the pixel. (3) This algorithm can improve the spatial resolution in a long time series based on the data of the LAI, and it also provides a method for regional variation analysis.

Based on the pixel scale, the algorithm proposed in this paper is based on the AVHRR NDVI and MODIS LAI to establish regression equations. The algorithm applied mixed pixel decomposition to improve the inversion of LAI spatial resolution. The algorithm improved the spatial resolution of the AVHRR LAI. Comparing this result with the MODIS LAI can satisfy the area within the regional scale of the study. This method is simple and convenient and contains fewer parameters for a long period. It can also be applied to other calculations of vegetation indices and analysis at the pixel scale.

## References

[CR1] Brown ME, Pinzon JE, Didan K (2006). Evaluation of the consistency of long-term NDVI time series derived from AVHRR, SPOT-vegetation, SeaWiFS, MODIS, and landsat ETM + sensors. IEEE Trans Geosci Remote Sens.

[CR2] Chen JM (1999). Spatial scaling of a remotely sensed surface parameter by contexture. Remote Sens Environ.

[CR3] Chen X, Wang H (2009). Spatial and temporal variations of vegetation belts and vegetation cover degrees in inner Mongolia from 1982 to 2003. Acta Geogr Sin.

[CR4] Chen JM, Pavlic G, Brown L (2002). Derivation and validation of Canada-wide coarse-resolution leaf area index maps using high-resolution satellite imagery and ground measurements. Remote Sens Environ.

[CR5] Deng F, Chen M, Plummer S (2006). Algorithm for global leaf area index retrieval using satellite imagery. IEEE Trans Geosci Remote Sens.

[CR6] Ganguly S, Samanta A, Schull MA (2008). Generating vegetation leaf area index Earth system data record from multiple sensors. Part 2: implementation, analysis and validation. Remote Sens Environ.

[CR7] Ganguly S, Schull MA, Samanta A (2008). Generating vegetation leaf area index earth system data record from multiple sensors. Part 1: theory. Remote Sens Environ.

[CR8] Holben BN (1986). Characteristics of maximum-value composite images from temporal AVHRR data. Int J Remote Sens.

[CR9] Huete AR (1988). A soil-adjusted vegetation index (SAVI). Remote Sens Environ.

[CR10] Jordan CF (1969). Derivation of leaf-area index from quality of light on the forest floor. Ecology.

[CR11] Liu R, Chen JM, Liu J (2007). Application of a new leaf area index algorithm to China’s landmass using MODIS data for carbon cycle research. J Environ Manag.

[CR12] Rouse JW, Haas RH, Schell JA, Deering DW (1974). Monitoring vegetation systems in the Great Plains with ERTS. NASA Spec Publ.

[CR13] Shabanov NV, Huang D, Yang W (2005). Analysis and optimization of the MODIS leaf area index algorithm retrievals over broadleaf forests. IEEE Trans Geosci Remote Sens.

[CR001] Tang S, Chen JM, Zhu Q (2007). LAI inversion algorithm based on directional reflectance kernels. J Environ Manag.

[CR14] Tucker CJ (2005). An extended AVHRR 8-km NDVI dataset comparable with MODIS and SPOT vegetation NDVI data. Int J Remote Sens.

[CR15] Yang W, Tan B, Huang D (2006). MODIS leaf area index products: from validation to algorithm improvement. IEEE Trans Geosci Remote Sens.

[CR16] Yang W, Shabanov NV, Huang D (2006). Analysis of leaf area index products from combination of MODIS Terra and Aqua data. Remote Sens Environ.

